# One-time root-zone N fertilization increases maize yield, NUE and reduces soil N losses in lime concretion black soil

**DOI:** 10.1038/s41598-018-28642-0

**Published:** 2018-07-06

**Authors:** Chaoqiang Jiang, Dianjun Lu, Chaolong Zu, Jia Shen, Shiji Wang, Zhibin Guo, Jianmin Zhou, Huoyan Wang

**Affiliations:** 1 State Key Laboratory of Soil and Sustainable Agriculture, Institute of Soil Science, Chinese Academy of Sciences, Nanjing, 210008 P. R. China; 2 Tobacco Research Institute/Maize Research Institute, Anhui Academy of Agricultural Sciences, Hefei, 230031 P. R. China; 3 Soil and Fertilizer Research Institute, Anhui Academy of Agricultural Sciences, Hefei, 230031 P. R. China

## Abstract

Excess N-fertilizer application and inappropriate fertilization methods have led to low N use efficiency (NUE) and high N leaching. A field experiment was performed in a typical lime concretion black soil area to compare N application methods: split surface broadcasting (SSB) and one-time root-zone fertilization (RZF) on grain yield, NUE, the fate of ^15^N urea and soil N loss during the 2015 and 2016 maize growing seasons. Each application method was tested at N rates of 135 and 180 kg N ha^−1^, and a control (CK) with no N fertilizer. The RZF treatment remarkably increased grain yield by 7.0% compared with SSB treatment under 180 kg N ha^−1^, and significantly increased N derived from fertilizer by 28.5%. The residual ^15^N in the 0–80 cm soil layer was 40.6–47.6% after harvest, 61.8–70.9% of which was retained in 0–20 cm. The RZF remarkably increased the ^15^N recovery in maize by 28.7%, while significantly decreased the potential N losses by 30.2% compared with SSB in both seasons. In conclusion, one-time RZF of urea is recommended for obtaining high yields, increasing NUE, and minimizing N losses in maize, which deserves more attention for developing and applying in the future.

## Introduction

Nitrogen (N) fertilization is one of the most important farming practices to improve grain yield of cereal crops^1^. High rates of N fertilizer, especially synthetic N fertilizer, are often applied to achieve high yields in China^1,2^; however, the increased fertilizer input has not resulted in consistently higher crop yields^3,4^. Instead, excess N fertilizer and poor fertilization methods have led to low N use efficiency (NUE) and high N losses^1,5^, resulting in a great number of environmental problems, such as water pollution, atmospheric contamination, and soil quality degradation^6,7^. Therefore, efficient N fertilizer management is vital to increase crop yields, improve soil fertility and minimize environmental risk^8–10^. Effective N management can improve NUE, and depends largely on the combination of appropriate sources, application rates, timing, and placement of N fertilizer application^11–13^. Numerous studies have been conducted to improve crop yield and NUE, and reduce N losses by adjusting the N application rate^5,14,15^. Many researchers have reported that under the same N application rate, splitting the number of N applications according to the plant’s N needs can reduce N losses and increase yield and NUE^14,16^. Moreover, using enhanced N fertilizers, such as urease inhibitors and controlled release fertilizer, have been shown to improve NUE and yields in rice, wheat and maize crops^17,18^. However, more labor or higher prices have limited the expansion of this alternative techniques^19–21^.

Increasing the N split application numbers according to the plant’s N needs was suggested for increasing NUE and reducing N losses^16,19^. Wang *et al*.^5^ reported that the N recovery efficiency for 2-split and 3-split application of urea was significantly higher than that for one-time mixed basal dressing, while the potential N loss was decreased remarkably. However, recently, Yao *et al*.^21^ found that point deep-placement at one time as a basal fertilizer significantly increased N recovery efficiency (NRE) by 55%, while decreased ammonia (NH_3_) volatilization by 91% compared to surface split broadcasting treatments. Grain yield and agronomic efficiency of fertilizer deep placement were superior to conventional farmer’s split method^19^. Therefore, split fertilization can be replaced by one-time basal fertilization only if the fertilization method was improved effectively. It has been widely recognized that one-time urea deep placement (UDP) can increase crop yields and reduce N losses^22–26^. We recently reported that root-zone fertilization (RZF) was effective in reducing N losses in the rice paddy fields^27^ and in the wheat–soil system^4^. RZF is a much more exact deep placement (DP) of fertilization according to the specific crop. For summer maize, we found that N applied all at one time as a basal fertilizer into a hole 5 cm away from the seed and 12 cm under the soil surface was considered to be a suitable RZF^28^. However, few studies have assessed the fate of N fertilizer under different application methods or the potential losses of N under RZF in a summer-maize dryland, specifically in the Huang-Huai-Hai Plain of China.

Huang-Huai-Hai Plain is one of the most important agricultural regions in China. The dominant cropping system is a rotation of summer-maize/winter-wheat^29,30^. One of the most important soil types in the region is the lime concretion black soil, also called Shajiang black soil, which plays an important role in food production^31^. The lime concretion black soil is found mainly in Huaibei Plain (located in the southern of the Huang-Huai-Hai Plain), which is characterized by heavy texture, low soil physical quality and soil fertility^31^. Lime concretion black soil was exhibited lower NUE in wheat than that of Chao soil, and lower N retention^32^, but showed a faster release of urea than that in fluvo-aquic soil^33^. However, limited information is available on the effect of urea application method on NUE in lime concretion black soil. The arable land in the Huang-Huai-Hai Plain is characterized by intense farming, with high rates of N fertilizer inputs and high crop yields^29^. In this region, the annual N fertilizer application for all crops amounts to 500 kg N ha^−1 ^^34^, and the N rate for maize has been reported to be 270 kg N ha^−1^, with a low NUE of about 28.5%^35^. However, in 2015, the Chinese Ministry of Agriculture issued Zero Increase Action Plan to curb the increase in fertilizer usage to zero by 2020^36^, which aims to increase crop yields without increasing fertilizer use, in an effort to reduce negative human impacts and environmental costs. To achieve this goal, it is imperative to improve NUE and reduce N losses, without further increase of N fertilizer use even decreases N application by 20%.

In maize production, N fertilizers are typically split into two applications in early June and late July, using the traditional surface broadcasting. The current practice of SSB N application is labor intensive and causes large amount of N to leach into the environment, yet it does not substantially increase maize yields^10,37^. Therefore, a better N management strategy based on reducing N losses and substantially increasing yields is needed. Our recent study showed that RZF has great promise for accomplishing both of these goals in the rice paddy fields^27,28,38^, while not effective in the winter wheat-soil system^4^. Although both the maize–soil system and wheat-soil system are dryland, the growth period of maize is about only 4 months, which is obviously shorter than that of wheat (nearly 8 months). Therefore, the fate of fertilizer N under the application of RZF in the dryland system, especially in the maize–soil system, should be played more attention. Here, we used ^15^N-labeled urea fertilizer to track the movement of N under two fertilization application methods, two-application SSB and one-time RZF, in a maize cropping system. A field experiment was conducted during two consecutive maize cropping seasons (2015 and 2016) in the Huang-Huai-Hai Plain of China.

## Results

### Maize yields

**Figure 1:**
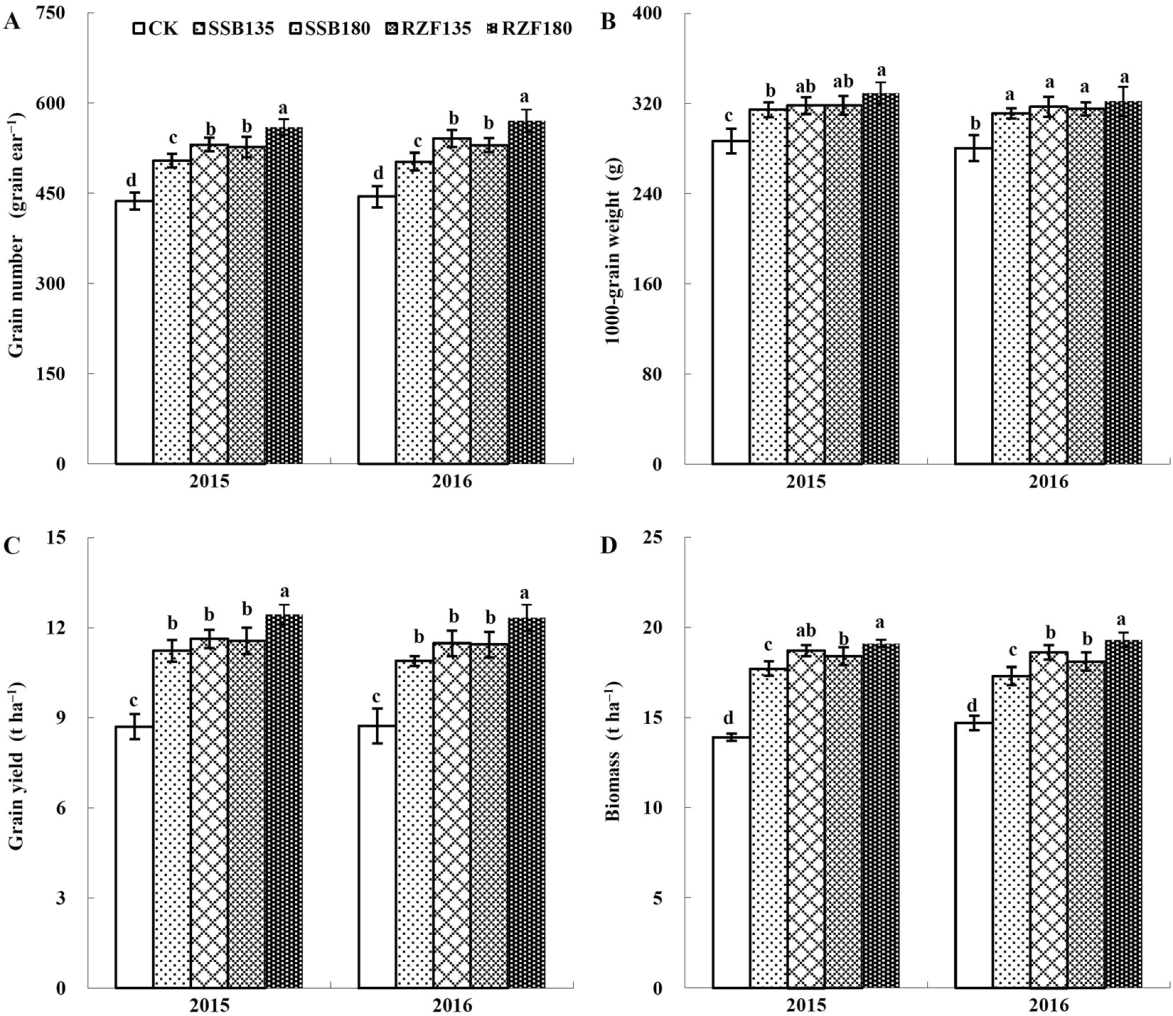
Effects of method of application of N and its dose on grain number, 1000-grain weight, grain yield and biomass of maize. CK: N application 0 kg ha^−1^, SSB135: two-split surface broadcasting with 135 kg N ha^−1^; SSB180: two-split surface broadcasting with 180 kg N ha^−1^; RZF135: one-time root-zone fertilization with 135 kg N ha^−1^; RZF180: one-time root-zone fertilization with 180 kg N ha^−1^. Columns labelled with different letters between treatments represent significant differences (*P* < 0.05).

N rate and application method significantly affected grain number per ear, grain yield and biomass of maize (Fig. 1). RZF of urea greatly increased grain number per ear of maize compared to surface broadcasting treatments. However, the 1000-grain weight of maize was not significantly affected by N application method in both 2015 and 2016. Grain yield of maize was significantly higher in the fertilizer treatments (10.9–12.4 t ha^−1^) than in the control (8.7 t ha^−1^) in both seasons. The grain yield was 6.9% and 7.0% higher in RZF180 compared with SSB180 in 2015 and 2016, respectively. However, there was no significant difference between SSB and RZF at low doses of N (135 kg ha^−1^) in both 2015 and 2016. The grain yield of RZF180 was significantly higher than that of RZF135, while they were not significantly different between SSB180 and SSB135. The biomass in RZF180 (19.3 t ha^−1^) was significantly higher than in SSB180 (18.6 t ha^−1^) in 2016.

### N concentration and N uptake by maize

The N concentration in both grain and straw were significantly affected by the applied doses of N, but not significantly affected by application method during two consecutive growing seasons (Table 1). In 2015, the concentrations of N in grains were significantly higher in RZF135 (13.4 g kg^−1^) and RZF180 (13.9 g kg^−1^) than that in SSB135 (12.8 g kg^−1^) and SSB180 (13.1 g kg^−1^), whereas there were no differences in 2016. There was no substantial difference in N concentration in straw between any of the fertilizer treatments (Table 1). N uptake by grain and straw and total N uptake by plant were all significantly affected by N dose. In both seasons, the N uptake in maize plants increased with increasing N application rate. Overall, the total N uptake by the whole plant in RZF was higher than that in SSB: by 5.8% and 4.5% in 2015 and 2016, respectively (Table 1).

**Table 1:** Maize plant nitrogen (N) concentration and rate of N uptake under two fertilization methods and two N rates over a two year period.

Treatments	N rate (kg ha^−1^)	2015					2016				
		N concentration (g kg^−1^)		N uptake (kg ha^−1^)			N concentration (g kg^−1^)		N uptake (kg ha^−1^)		
		Grain	Straw	Grain	Straw	Total	Grain	Straw	Grain	Straw	Total
CK	0	11.4 c	10.9 a	88.9 c	63.4 b	152.4 c	13.3 b	11.7 b	96.6 b	84.8 b	181.4 c
SSB	135	12.8 b	12.2 a	136.1 b	85.9 a	222.0 b	14.7 a	13.4 a	140.8 a	103.7 ab	244.5 b
	180	13.1 ab	12.4 a	144.0 ab	90.7 a	234.7 ab	14.9 a	13.6 a	145.5 a	113.3 a	258.7 a
RZF	135	13.4 ab	12.6 a	146.9 a	87.2 a	234.1 ab	14.7 a	13.7 a	143.5 a	110.9 a	254.5 ab
	180	13.9 a	12.6 a	154.5 a	94.3 a	248.9 a	15.0 a	13.8 a	153.8 a	117.8 a	271.6 a
Rate (R)		***	*	***	***	***	***	***	***	**	***
Method (D)		ns	ns	*	ns	*	ns	ns	ns	ns	ns
R × D		ns	ns	ns	ns	ns	ns	ns	ns	ns	ns

Values followed by different letters in the same column represent significant differences (*P* < 0.05). CK: N application 0 kg ha^−1^; SSB: two-split surface broadcasting; RZF: one-time root-zone fertilization. ns means not significant. *Significant at *P* < 0.05; **Significant at *P* < 0.01; ***Significant at *P* < 0.001.

### Nitrogen-use efficiency

The N apparent recovery efficiency (NARE), N agronomy efficiency (NAE) and N partial factor productivity (NPFP) of maize over two consecutive growing seasons are shown in Table 2. The application dose of N had a significant effect on NAE and NPFP, but did not affect NARE. The NAE and NPFP were significantly higher at 135 kg N ha^−1^ treatments (23.2 and 86.7 kg kg^−1^, respectively) than that of 180 kg N ha^−1^ treatments (19.0 and 66.5 kg kg^−1^). The N application method had a significant effect on NARE, but did not affect NPFP over two consecutive growing seasons; and also affected the NAE in 2015, but did not significantly affect the NAE in 2016. Compared with the SSB, RZF significantly increased NARE by 14.1% and 19.4% at 135 and 180 kg N ha^−1^, respectively.

**Table 2:** Effects of method of application of N and its dose on N use efficiency.

Treatments	N rate (kg ha^−1^)	2015			2016		
		NARE (%)	NAE (kg kg^−1^)	NPFP (kg kg^−1^)	NARE (%)	NAE (kg kg^−1^)	NPFP (kg kg^−1^)
SSB	135	51.6 a,b	24.5 a	87.9 a	46.8 ab	20.0 a	83.4 a
	180	45.7 b	19.5 b	67.1 b	41.2 b	16.4 b	63.9 b
RZF	135	60.6 a	25.8 a	89.3 a	51.8 a	22.4 a	86.1 a
	180	53.6 ab	20.2 b	67.8 b	50.1 a	19.7 ab	67.2 b
Rate (R)		ns	**	***	ns	*	***
Method (D)		*	ns	ns	*	*	ns
R × D		ns	ns	ns	ns	ns	ns

Values followed by different letters in the same column represent significant differences (*P* < 0.05). SSB: two-split surface broadcasting; RZF: one-time root-zone fertilization. ns means not significant. *Significant at *P* < 0.05; **Significant at *P* < 0.01; ***Significant at *P* < 0.001.

### Sources of plant N uptake and ^15^N distribution in maize

N derived from fertilizer (Ndff) was significantly affected by N application method, N dose, and interaction between N application method and dose in 2015 and 2016, whereas neither N application method nor N dose significantly affected the N derived from soil (Ndfs) (Table 3). In both seasons, the Ndff at 180 kg N ha^−1^ treatments was 21.3% higher than that of 135 kg N ha^−1^ treatments. In RZF treatments, the Ndff increased from 17.5% to 22.9% on average when the N application rate increased form 135 to 180 kg ha^−1^. Moreover, the Ndff was 28.5% higher on average in RZF compared with SSB in 2015 and 2016.

**Table 3:** Maize plant N derived from fertilizer (Ndff) and N derived from soils (Ndfs).

Treatments	N rate (kg ha^−1^)	2015			2016		
		Ndff (kg ha^−1^)	Ndfs (kg ha^−1^)	Ndff (%)	Ndff (kg ha^−1^)	Ndfs (kg ha^−1^)	Ndff (%)
SSB	135	31.9 c	199.7 a	13.8 c	43.5 c	211.7 a	17.1 c
	180	36.2 b	209.2 a	14.7 bc	55.0 b	217.7 a	20.2 b
RZF	135	38.0 b	206.1 a	15.6 b	51.5 b	214.3 a	19.4 bc
	180	50.4 a	209.3 a	19.4 a	75.5 a	211.6 a	26.4 a
Rate (R)		***	ns	***	***	ns	***
Method (D)		***	ns	***	***	ns	***
R × D		***	ns	*	*	ns	*

Values followed by different letters in the same column represent significant differences (*P* < 0.05). SSB: two-split surface broadcasting; RZF: one-time root-zone fertilization. ns means not significant. *Significant at *P* < 0.05; **Significant at *P* < 0.01; ***Significant at *P* < 0.001.

**Figure 2:**
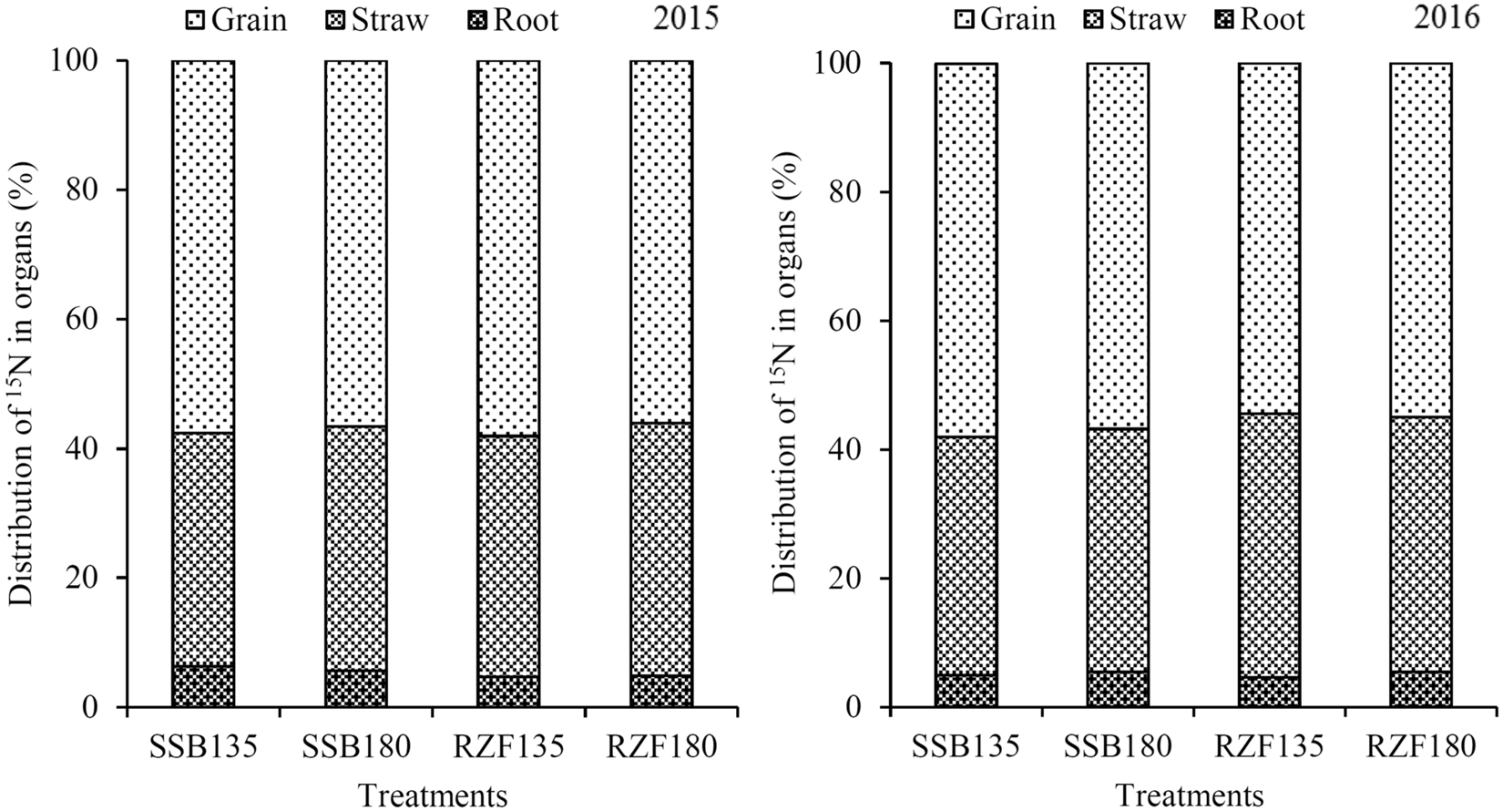
Distribution of 15N-labeled urea in maize organs as a factor of application dose (135 or 180 kg N ha^−1^) and application method (root zone fertilization, RZF, or split surface broadcast, SSB) in two consecutive growing seasons (2015–2016). SSB135: two-split surface broadcasting with 135 kg N ha^−1^; SSB180: two-split surface broadcasting with 180 kg N ha^−1^; RZF135: one-time root-zone fertilization with 135 kg N ha^−1^; RZF180: one-time root-zone fertilization with 180 kg N ha^−1^.

Neither N application method nor N dose significantly affected the distribution of ^15^N among different organs of maize, except that ^15^N uptake in roots was significantly higher in the high N application method in 2015 (Fig. 2). The distribution of ^15^N among different organs was as follows: grain > straw > root. In 2015 and 2016, 56.1–58.1% and 54.5–58.0%, respectively, of total ^15^N uptake by the plant was partitioned to grain. Overall, the distribution of ^15^N in grain, straw and root did not significantly changed by N application method or N dose.

### Distribution of residual ^15^N-labeled urea in soil

**Figure 3:**
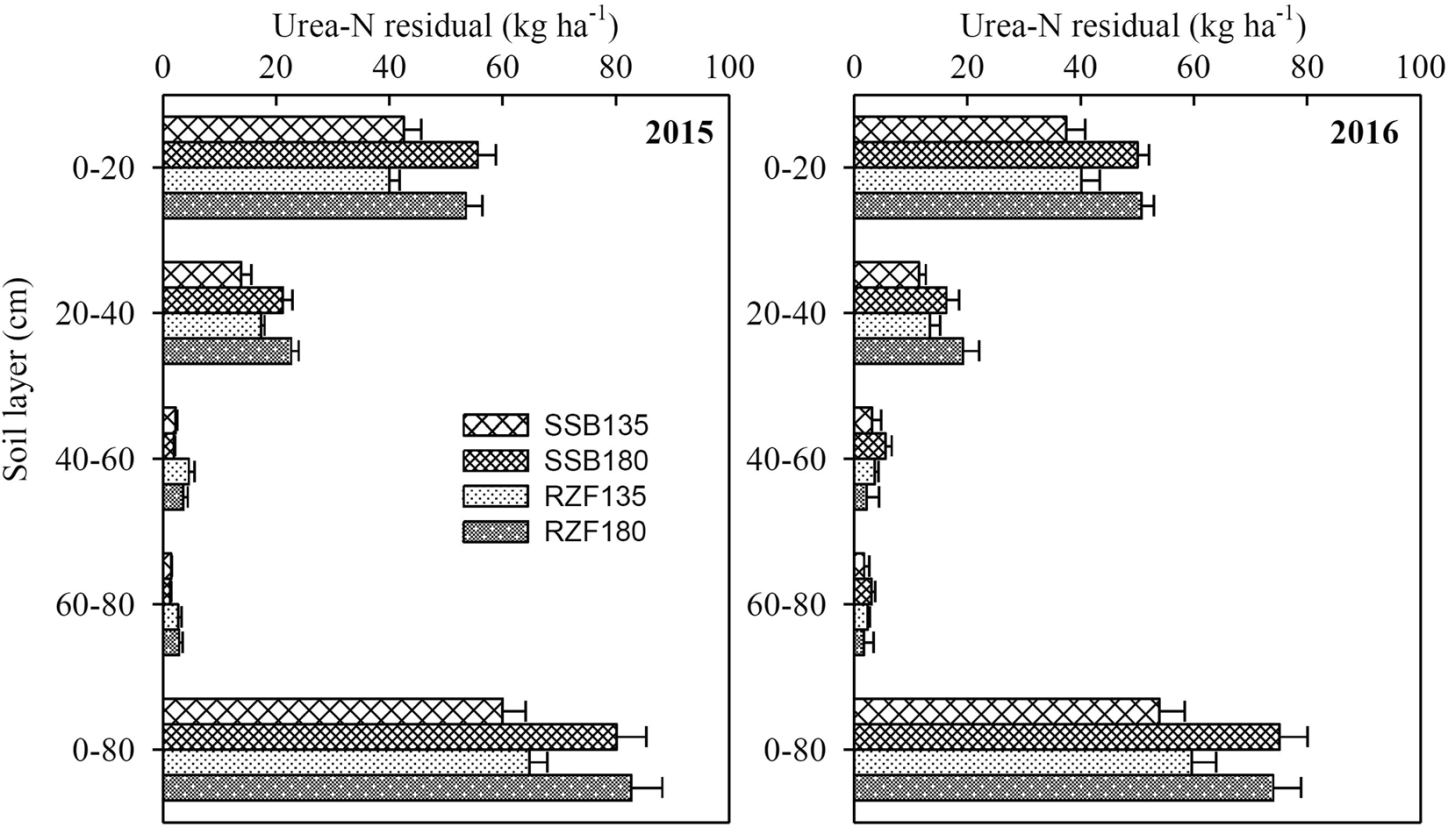
The distribution of residual 15N at the end of each harvest season as a factor of application method and rate of N. SSB135: two-split surface broadcasting with 135 kg N ha^−1^; SSB180: two-split surface broadcasting with 180 kg N ha^−1^; RZF135: one-time root-zone fertilization with 135 kg N ha^−1^; RZF180: one-time root-zone fertilization with 180 kg N ha^−1^.

The pattern of residual ^15^N distribution in the soil among treatments was not different for either growing seasons (Fig. 3). The total residual ^15^N in the 0–80 cm soil profile layer after maize harvest was 54.8–64.3 kg ha^−1^ in the two growing season under 135 kg N ha^−1^ treatments, accounting for 40.6–47.6% of ^15^N, and 73.8–81.7 kg ha^−1^ under 180 kg N ha^−1^ treatments, accounting for 41.0–45.4% of ^15^N, with about two-thirds (67.3%) of the residual ^15^N remaining in the 0–20 cm soil layer. The total residual ^15^N in the 0–80 cm soil profile layer was significantly affected by neither N application method nor N dose. However, the distribution of residual ^15^N in soil profile layer was significantly affected by the N application method, especially in 0–20 cm soil layer. The percentage of residual ^15^N in 0–20 cm soil layer was slightly higher in SSB (69.1%) than that in RZF (65.7%).

### Fate of ^15^N-labeledled urea in maize–soil system

The N recovery and potential losses were significantly affected by N application method, whereas the residual was affected by neither N application method nor N dose over two consecutive growing seasons (Table 4). In RZF, the recovery of ^15^N-labeled urea in maize was 28.2–38.2% at 135 kg N ha^−1^ treatments and 28.0–42.0% at 180 kg N ha^−1^ treatments. Therefore, the N dose did not significantly affected the fate of ^15^N-labeled urea in maize growing seasons. On average, the recovery of N was 28.7% higher in RZF than that in SSB over two years. The potential N losses were 17.1–26.2% in RZF and 27.2–35.7% in SSB. Overall, the 2-year average potential N losses were 30.2% lower in RZF than that in SSB.

**Table 4:** The fate of ^15^N-labeled urea in both maize growing seasons.

Treatments	N rate (kg ha^−1^)	2015			2016		
		Recovery in maize (%)	Residual in soil (%)	Potential losses (%)	Recovery in maize (%)	Residual in soil (%)	Potential losses (%)
SSB	135	23.6 b	44.2 a	32.2 ab	32.2 b	40.6 a	27.2 a
	180	20.1 c	44.2 a	35.7 a	30.6 b	42.0 a	27.5 a
RZF	135	28.2 a	47.6 a	24.2 c	38.2 a	43.6 a	18.3 b
	180	28.0 a	45.4 a	26.6 bc	42.0 a	41.0 a	17.1 b
Rate (R)		**	ns	ns	ns	ns	ns
Method (D)		***	ns	**	***	ns	**
R × D		**	ns	ns	*	ns	Ns

Values followed by different letters in the same column represent significant differences (*P* < 0.05). SSB: two-split surface broadcasting; RZF: one-time root-zone fertilization. ns means not significant. *Significant at *P* < 0.05; **Significant at *P* < 0.01; ***Significant at *P* < 0.001.

## Discussion

In the present study, the grain yield was 7.0% higher in RZF180 compared with SSB180 in both seasons. Chen *et al*.^14^ reported a mean maize yield of about 13.0 t ha^−1^ at N rate of 237 ± 70 kg ha^−1^ on 66 on-farm experimental plots under an integrated soil–crop system management. Moreover, the maize grain yield reached 14.8 t ha^−1^ at 250 kg N ha^−1^ under a high-yield maize system in northern China^39^. However, the maize yield in this study was significant higher than that of another study in Huang-Huai-Hai Plain of China, which reported that the maize grain yield was only 9.0 t ha^−1^ under the N application rate of 225 kg ha^−1 ^^30^. Yang *et al*.^40^ also reported that grain yield of summer maize was 10.5 t ha^−1^ at 180 kg N ha^−1^ in North China Plain of China. Therefore, both the RZF180 and SSB180 treatments in the present study could be classified as “high” yields of summer maize. However, RZF of urea lead to higher grain number per ear and aboveground biomass which have a positive relationship with yield compared with surface broadcasting. Therefore, one-time RZF of urea achieved higher grain yield and biomass than 2-split surface broadcasting for summer maize system in this study.

Previous studies have reported that deep placement of N significantly increases N uptake by crop plants^27,41,42^. Rees *et al*.^41^ found that N uptake by maize under N deep placement increased by 7.9–32.5% compared with surface broadcasting. We also found that one-time urea RZF can promote N uptake by rice and thus increase grain yield, compared with three-split surface broadcasting treatments^27^. These results agree with the present study; we found that N placement significantly affected the N uptake in grain and the whole plant. Although there was interannual variability due to differences in weather conditions (mainly in rainfall), the N uptake by maize was substantially higher in RZF than SSB treatments in both 2015 and 2016 (Table 1). Compared with surface broadcasting, deep placement of urea can provide more N for the deep regions of the rhizosphere, thus improving the growth of roots and N uptake by plants^43^. Moreover, deep placement of N fertilizer prolonged N availability up to flowering^19^, and increased the number of tillers and panicles in rice^27,44^.

Fertilizer placement had a significant effect on N uptake from fertilizer. The Ndff was 18.4% and 39.2% higher in RZF than SSB for 135 and 180 kg N ha^−1^ treatments, respectively, over two consecutive growing seasons (Table 3). These results are in agreement with previous work that found that point placement significantly increases the Ndff (%) by 30.4% compared with surface application, in summer maize^41^. Yao *et al*.^21^ also reported that N uptake by rice derived from fertilizer was 62% higher in one-time UDP treatment than that in surface broadcasting. The higher Ndff in RZF may be attributed to point deep placement significantly increasing the nutrient content in the root zone^27,43^ and decreasing N losses through runoff and NH_3_ volatilization, compared with surface applications^43,45^. In addition, the Ndff (%) was higher in the higher N application rate treatments for both RZF and SSB. The Ndff (%) under 180 kg N ha^−1^ treatments increased by 30.2% and 12.3% compared with 135 kg N ha^−1^ treatments in RZF and SSB, respectively (Table 3). This is consistent with previous studies by Yang *et al*.^40^, who found that the Ndff (%) in the total maize plant increased from 23.7% to 43.6% with increasing N application rate from 90 to 135 kg N ha^−1^. Chen *et al*.^46^ also reported that the Ndff (%) of winter wheat increased from 34.6% to 41.8%, when the N dose increased from 150 to 240 kg ha^−1^. In addition, the suitable N fertilization rate for the summer maize in Huang-Huai-Hai Plain^47^ and the lime concretion black soil region^48^ were 113–180 and 144–225 kg ha^−1^, respectively. Overall, these results suggest that the rate of 135 kg N ha^−1^ is not sufficient for maize plants, while the application rate of 180 kg N ha^−1^ may be generally suitable for obtaining high yields and maintaining low potential N losses for maize grown in lime concretion black soils of the Huang-Huai-Hai Plain. The present study suggests that one-time RZF can further reduce the amount of N fertilizer, which deserves more attention and further research in the future.

In the present study, the NUE (NARE and N recovery) of RZF were significantly higher than that of SSB (Tables 2 and 4). High NUE obtained with deep placement agrees with the findings from other studies^41,49,50^. For example, Rees *et al*.^41^ reported that the NRE of summer maize under deep placement increased from 18% to 25% compared with surface application. Similarly, Yao *et al*.^21^ found that one-time UDP significantly increased NARE by 52.6% during three consecutive rice cropping seasons compared with 3-split surface broadcasting. Therefore, this study suggests that N point deep placement in the root-zone can increase N recovery in maize and improve NUE.

The potential N losses in all treatments were 17.2–35.7% (Table 4), consistent with previous reports. Yang *et al*.^40^ found 14–33% N losses in summer maize in North China Plain. Ju *et al*.^51^ reported that total N fertilizer losses for summer maize were 21.1–51.3% in the North China Plain, with a higher potential N loss at the conventional application rate (360 kg N ha^−1^). However, the potential N losses of this study were higher than that of maize in semiarid farmland reported by Wang *et al*.^5^ (averaged 15.4% among all treatments). The lower potential N losses reported by Wang *et al*.^5^ may be attributed to the plastic mulching practice, which could decrease N leaching losses and result in higher levels of ^15^N-labeled remaining in the 0–170 cm soil profile^5,43^. Compared with surface broadcasting, root-zone fertilization greatly reduced total potential N losses. In both seasons, the potential N losses were 17.1–26.2% in RZF, which was 30.2% lower than that in SSB (27.2–35.7%) (Table 4). This is consistent with Cai *et al*.^45^, which reported that total N loss for maize in deep placement decreased from 54.5% to 18.5% compared with surface broadcasting. Similarly, the ^15^N loss was 38% lower for one-time deep placement of urea compared with three-split surface broadcasting^21^. Deep placement of urea enormously reduced both NH_3_ volatilization and denitrification losses compared with split surface broadcasting^43,45^, probably as a result of better preservation and concentration of N fertilizer beneath plant roots and the reduction of soil microbes competing with plants for the point deep-placed N^21,27^. In contrast, when urea was broadcast onto the soil surface, it was easily lost through NH_3_ volatilization and runoff^21^. Therefore, as a much more exact deep placement of fertilization, the RZF of urea could substantially reduce ammonia volatilization, denitrification loss and runoff for maize systems in lime concretion black soil of the Huang-Huai-Hai Plain. In general, RZF is a fertilizer-saving and N-loss reducing fertilization method, but it is worth developing and applying the special root-zone fertilization machinery.

RZF of urea did not affect the total residual ^15^N in the 0–80 cm soil profile layer. On average, the residual ^15^N-labeled fertilizer in the 0–80 cm soil layer was 59.6 and 78.0 kg ha^−1^ under 135 and 180 kg N ha^−1^ treatments, respectively, accounting for 44% and 43% of the total ^15^N application (Fig. 3 and Table 4). This result is supported by that obtained for maize in North China Plain by Yang *et al*.^40^, who found that 45–60% of the applied N remained in the 0–150 cm soil layer at the first maize harvest. Wang *et al*.^5^ also found that the residual ^15^N in the 0–200 cm soil layer was about 48.3–51.3% at harvest in semiarid plastic mulched maize cropping system. Moreover, we found that two-thirds (61.8–70.9%) of the residual ^15^N was retained in the 0–20 cm layer (Fig. 3). Yang *et al*.^40^ and Wang *et al*.^5^ reported that approximately half of the residual N remained in the 0–20 cm layer at the summer-maize harvest. However, in our previous study, 76.8–87.0% of total residual N remained in the 0–20 cm soil layer for winter wheat in south-eastern China^46^. The difference may be due to differences in soil properties; in Yang *et al*.^40^, the soil in the test site was a sandy loam, which may increase the risk of N leaching compared with the loamy soil in the Chen *et al*.^46^ experiment. Moreover, the point deep placement can reduce nitrate and total N leaching losses^21,52^, thus more residual ^15^N was retained in the topsoil (0–20 cm). Therefore, our results suggest that RZF may be an effective approach to further reduce N loss through leaching for summer maize, especially with high rainfall and course-textured or sandy soils.

In conclusion, one-time RZF of urea (180 kg N ha^−1^) achieved 7.0% higher grain yield than SSB treatment. RZF significantly increased the Ndff in maize plants by 28.5% compared with SSB. The residual ^15^N in the 0–80 cm soil layer ranged from 40.6% to 47.6% at maize harvest, approximately two-thirds (61.8–70.9%) of which was retained in the 0–20 cm soil layer. RZF significantly enhanced NARE and N recovery in maize compared with SSB in both seasons, while significantly decreased the potential N losses by 30.2%. Overall, our study suggests that one-time RZF of urea is a superior N application method for obtaining high-yield levels, increasing NUE, and minimizing soil N losses in the maize cropping system.

## Materials and Methods

### Experimental site

**Figure 4:**
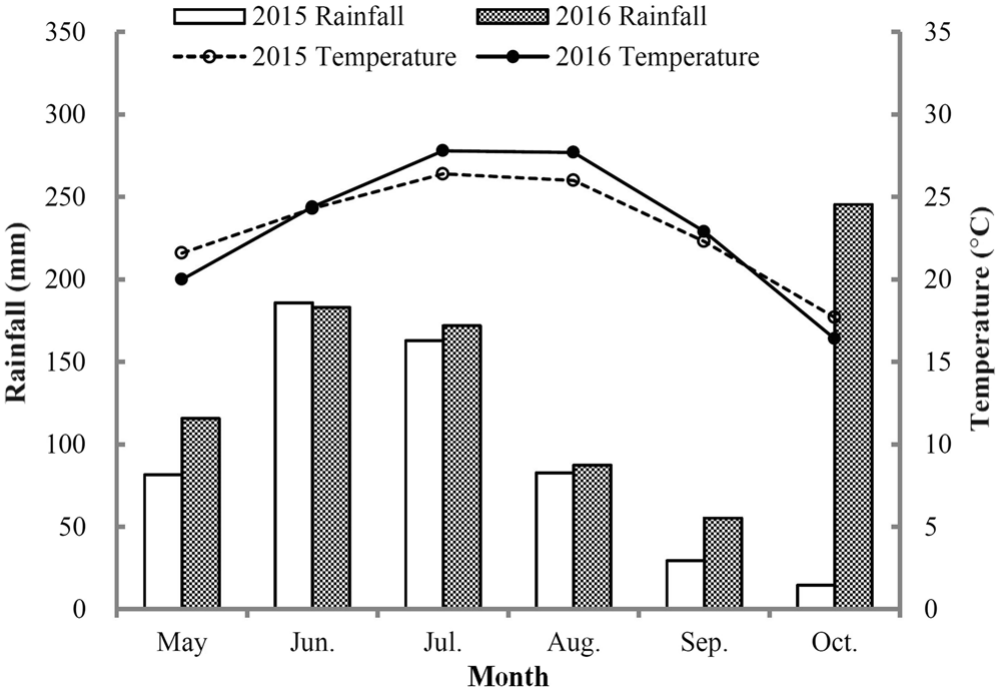
Monthly mean rainfall and temperature during the maize growing season in the Huang-Huai-Hai Plain of China.

The experiment was performed in a farmer’s field adjacent to the research station of the Anhui Academy of Agricultural Sciences (AAAS) in Taihe County (33°15′N, 115°36′E), Anhui province, China. The soil of the experimental site is classified as a typical lime concretion black soil. Before the experiment in June 2015, the physicochemical properties of the topsoil (0–20 cm) are pH 8.0 (H_2_O), 18.5 g kg^−1^ organic matter, 1.33 g kg^−1^ total N, 90.6 mg kg^−1^ available N, 22.6 mg kg^−1^ available P, and 250.4 available K, 1.30 g cm^−3^ soil bulk density. The study site is described as a warm temperate and semi-humid monsoon climate, with a mean temperature of 14.9 °C and mean annual precipitation of 900 mm (from 1953 to 2009). The mean monthly rainfall and air temperature during the experimental period in 2015 and 2016 is shown in Fig. 4.

### Experiment design and agricultural management practices

As the common in this area, summer-maize/winter-wheat rotation was used in this study. The field plot experiment was conducted for the two consecutive maize cropping seasons (2015–2016). Five treatments were assigned to the field plots: (1) a control with no N fertilizer (CK); (2) two-split surface broadcasting (SSB) with 135 kg N ha^−1^ (SSB135); (3) two-split surface broadcasting with 180 kg N ha^−1^ (SSB180); (4) one-time RZF with 135 kg N ha^−1^ (RZF135); and (5) one-time RZF with 180 kg N ha^−1^ (RZF180). The rates of N were selected according to the effect of different N rates (0, 90, 135, 180, 225, 270, and 360 kg N ha^−1^) on maize yield in our preliminary study (data no showed), and the previous studies carried out in Huang-Huai-Hai Plain^47^ and the lime concretion black soil region^48^, which recommended that the suitable N fertilization rate were 113–180 and 144–225 kg ha^−1^, respectively. The rates and timing of fertilizer application for the five treatments are shown in Table 5.

**Table 5:** Nitrogen application date and rate of different treatments.

Code	Application method	N rate (kg N ha^−1^)	Basal fertilization (kg N ha^−1^)	Topdressing (kg N ha^−1^)
CK		0	0	0
SSB135	2-split broadcasting	135	67.5	67.5
SSB180	2-split broadcasting	180	90	90
RZF135	Root-zone fertilization	135	135	0
RZF180	Root-zone fertilization	180	180	0
Application date			Basal fertilization	Topdressing
2015			Jun. 17	Aug. 2
2016			Jun. 15	Aug. 1

CK: N application 0 kg ha^−1^, SSB135: two-split surface broadcasting with 135 kg N ha^−1^; SSB180: two-split surface broadcasting with 180 kg N ha^−1^; RZF135: one-time root-zone fertilization with 135 kg N ha^−1^; RZF180: one-time root-zone fertilization with 180 kg N ha^−1^.

The five treatments were arranged in a randomized complete block design with three replicate plots of each treatment for a total of 15 plots each year. Plots were 2.4 m (4 rows) × 2.8 m length (40 plants for each plot) in 2015 and 3.0 m (5 rows) × 5.6 m length (100 plants for each plot) in 2016. To monitor the fate of ^15^N-labeled fertilizer, three plants were used within each plot, which were in the center of each main plot. Each main plot was separated by earthen banks (30 cm-wide and 50 cm-high) to prevent lateral water and nutrient inference, and each ^15^N fertilized plant was bordered by polyvinyl chloride (PVC) frame (50 cm-high, 28 cm-wide and 60 cm-long; open at the top and bottom) inserted into the soil to a depth of 45 cm. The maize cultivar was *Zea mays* L., cv ‘Longping 206’, a local prevailing cultivar, and the maize seeds were sowed at a spacing of 60 cm × 28 cm (60000 plant ha^−1^) in all treatments. The ^15^N-labeled urea (46% N content, and 10.15% of ^15^N abundance ratio) used in this experiment was provided by the Shanghai Research Institute of Chemical Industry. The non-labeled N fertilizer was urea (46% N). The phosphorus (P) fertilizer (135 kg P_2_O_5_ ha^−1^, superphosphate) and potassium (K) fertilizer (180 kg K_2_O ha^−1^, potassium chloride) were applied to were broadcast as basal fertilizers in all treatments at sowing. In SSB, 50% N fertilizer was broadcast by hand at sowing, and 50% at the tasseling stage. In the RZF treatment, the N fertilizer was point deep-placed all at one time as a basal fertilizer (4.89 and 6.52 g urea plant^−1^ for RZF135 and RZF180, respectively) into a hole 5 cm away from the seed and 12 cm under the soil surface, and the fertilization point was marked immediately.

Irrigation, pesticide and herbicide applications were the same for all treatments. The irrigation was applied twice (at the bell and grain-filling stages) by sprinklers during the maize growing season. For each time, sprinkler irrigation was applied until the soil surface was moist. Maize was sown by hand on June 17, 2015 and June 15, 2016, and harvested on September 27, 2015 and September 25, 2016.

### Plant and soil sampling and analysis

At maturity, grain yield and biomass were determined by harvesting all plants in each plot. Grain number per plant and 1000-grain weight was also measured for each plot. The ^15^N labelled plants in each plot were harvested close to the ground, and divided into grain and straw. And a total of 45 plant roots of the ^15^N labelled plants from the 0–60 cm soil layer were collected and washed. The fresh grain, straw, and root samples were dried at 70 °C before being ground to powder and passed through a 0.15-mm screen for N content and ^15^N analysis.

Soils were sampled in a 20-cm radius around each plant to a depth of 80 cm at 20-cm interval. Three plants were randomly selected for soil sample. Therefore, 12 soil samples were taken for each treatment, and a total of 48 soil samples were obtained. The soil samples were air-dried, then ground through 0.15-mm sieve. Soil bulk density was determined after harvesting the wheat using the cutting ring method^53^. Grain, straw, root, and soil samples were analyzed for total N and ^15^N abundance using an elemental analyzer (Costech ECS4010, Costech Analytical Technologies Inc., Valencia, USA) coupled to an isotope ratio mass spectrometer (Delta V Advantage, Thermo Fisher Scientific Inc., USA).

### Calculation methods

All ^15^N was expressed as the atom percent excess corrected for background abundance (i.e. 0.366%). The percentage of N derived from fertilizer (Ndff) was calculated according to the following equation^46,54^:

1 $${\rm{Ndff}}\,( \% )=\frac{{\rm{B}}-{\rm{A}}}{{\rm{C}}-{\rm{A}}}\times 100$$

where A is the ^15^N natural abundance, B is the ^15^N atom percent excess in the plant or soil, and C is the ^15^N atom percent excess in the fertilizer N.

The amounts of plant N derived from fertilizer (Ndff) and from soil (Ndfs), and the soil residual N were calculated in López-Bellido *et al*.^54^.

The N fertilizer accumulation and recovery by maize were calculated according to López-Bellido *et al*.^54^ and Wang *et al*.^5^ using the following equations:

2 $${\rm{Plant}}\,{\rm{total}}\,{\rm{N}}\,({\rm{kg}}\,{{\rm{ha}}}^{-{\rm{1}}})={\rm{plant}}\,{\rm{dry}}\,{\rm{matter}}\times {\rm{N}}\,{\rm{concentration}}$$

3 $${\rm{Plant}}\,{\rm{N}}\,{\rm{derived}}\,{\rm{from}}\,{\rm{fertilizer}}\,({\rm{Ndff}})\,({\rm{kg}}\,{{\rm{ha}}}^{-{\rm{1}}})=({\rm{2}})\times {{\rm{Ndff}}}_{{\rm{plant}}}$$

4 $${\rm{Soil}}\,{\rm{residual}}\,{\rm{N}}\,({\rm{kg}}\,{{\rm{ha}}}^{-{\rm{1}}})={\rm{N}}\,{\rm{concentration}}\times {{\rm{Ndff}}}_{{\rm{soil}}}\times {\rm{soil}}\,{\rm{bulk}}\,{\rm{density}}\times {\rm{soil}}\,{\rm{thickness}}$$

5 $${\rm{N}}\,{\rm{recovery}}\,{\rm{efficiency}}\,({\rm{NRE}})\,( \% )=({\rm{3}})/{\rm{N}}\,{\rm{application}}\,{\rm{rate}}\times {\rm{100}}$$

6 $${\rm{N}}\,{\rm{residual}}\,{\rm{efficiency}}\,( \% )=({\rm{4}})/{\rm{N}}\,{\rm{application}}\,{\rm{rate}}\times {\rm{100}}$$

7 $${\rm{Potential}}\,{\rm{N}}\,{\rm{losses}}\,( \% )={\rm{100}}\,\mbox{--}\,({\rm{5}})\,\mbox{--}\,({\rm{6}})$$

The NUE indexes, namely NARE, NAE and NPFP were calculated by following the method described by López-Bellido *et al*.^54^ and Liu *et al*.^43^.

### Statistical analysis

Statistical analysis was conducted using SPSS 19.0 for analysis of variance (ANOVA). Two-way ANOVA was conducted to assess the effects of N fertilizer placement and N rate on maize yield, N uptake and fate of ^15^N-labeled urea. Treatments were compared by the least significance difference at *α* < 0.05.
